# Cost-effectiveness of massed versus spaced trauma-focused treatment as first-line treatment for post-traumatic stress disorder in adults with multiple trauma exposure: protocol for a single-blind non-inferiority randomised controlled trial

**DOI:** 10.1136/bmjopen-2025-102530

**Published:** 2025-05-23

**Authors:** Bram Kemmere, Ytje T van Pelt, Miriam J J Lommen, Rafaele J C Huntjens, Miranda Olff, Mayaris Zepeda Méndez, Suzy Matthijssen, Leona Hakkaart-van Roijen, Mirjam J Nijdam, Foske Jackie June ter Heide

**Affiliations:** 1 Psychiatry, Amsterdam UMC Locatie AMC, Amsterdam, The Netherlands; 2 ARQ National Psychotrauma Centre, Diemen, The Netherlands; 3 Clinical Psychology and Experimental Psychopathology, University of Groningen, Groningen, The Netherlands; 4 Psy-Zo!, Groningen, The Netherlands; 5 University of Groningen, Groningen, The Netherlands; 6 Department Trauma Center, GGZ Drenthe Mental Health Institute, Beilen, Netherlands; 7 PSYTREC, Bilthoven, The Netherlands; 8 Erasmus Universiteit Rotterdam Erasmus School of Health Policy and Management, Rotterdam, The Netherlands

**Keywords:** Randomized Controlled Trial, Adult psychiatry, Psychosocial Intervention, Psychological Stress

## Abstract

**ABSTRACT:**

**Introduction:**

Post-traumatic stress disorder (PTSD) is a serious disorder that burdens individuals and society. The current standard of first-line treatment for PTSD is spaced trauma-focused treatment (S-TFT), involving weekly sessions. While effective, S-TFT may take relatively long to complete, especially in patients exposed to multiple potentially traumatic events (PTEs). Massed trauma-focused treatment (M-TFT), involving increased session frequency, potentially results in faster symptom reduction and restoration of quality of life, as well as in a reduction of societal costs. However, M-TFT is not recommended as first-line treatment. This paper describes the research protocol of a single-blind, multicentre randomised controlled trial (RCT) aimed at investigating: (1) the clinical and cost-effectiveness of M-TFT versus S-TFT in employed, multiply traumatised patients who seek first-line treatment for PTSD and (2) predictive and moderating factors related to treatment response.

**Methods and analysis:**

186 participants are recruited from five centres and will be included if they are ≥18 years old, meet criteria for a Diagnostic and Statistical Manual of Mental Disorders Fifth Edition PTSD diagnosis based on ≥two PTEs, seek treatment for the first time and are employed. Patients with specified comorbid disorders and insufficient Dutch language proficiency are excluded. Participants are randomised to 800 min of either M-TFT or S-TFT. M-TFT consists of two once-weekly preparatory sessions, 10 twice-daily sessions of prolonged exposure, eye movement desensitisation and reprocessing therapy for 2 weeks and two once-weekly closing sessions. S-TFT consists of weekly sessions of one of five evidence-based treatment interventions. Outcomes are assessed at baseline and at 7 weeks, 17 weeks, 6 months and 9 months after baseline. Primary outcomes are clinical effectiveness in terms of PTSD symptom severity and cost-effectiveness based on quality of life measures and societal costs. Data will be analysed with linear mixed models.

**Ethics and dissemination:**

This study protocol was approved by the Medical Ethics Review Board of the Amsterdam University Medical Center (NL86057.018.24). Participants will provide informed consent before enrolment in the trial. Results will be published in peer-reviewed journals and will be released to clinicians, patient groups and the general community.

**Trial registration number:**

This protocol is registered at Overview of Medical Research in the Netherlands (OMON; trial register number 56960) and ClinicalTrials.gov (NCT06700590).

**STRENGTHS AND LIMITATIONS OF THIS STUDY**

- With the inclusion of 186 participants, this randomised controlled trial (RCT) will be well-powered to establish the non-inferiority of massed trauma-focused treatment (M-TFT) compared with spaced-TFT (S-TFT).
- The current RCT will be conducted at five centres throughout the Netherlands, which will enrol patients with a variety of trauma backgrounds, increasing the generalisability of the findings.
- Assessments will be performed at five time points from baseline to 9-month follow-up, which allows for accurate investigation of the (cost-)effectiveness of the treatments over time.
- Elements of pragmatic, real world RCTs are present in the S-TFT condition in which multiple types of evidence-based trauma treatment are allowed, enabling a more representative comparison of M-TFT to standard clinical care.
- A potential limitation concerns differences between the M-TFT condition and the S-TFT condition in terms of therapist rotation, supervision frequency and the alternation of therapeutic elements.

## Introduction

 Post-traumatic stress disorder (PTSD) is a severe psychiatric disorder characterised by intrusive memories, avoidance, negative alterations in cognition and mood and hyperarousal.^1^ It is associated with psychiatric and somatic comorbidity such as depression and chronic pain, reduced quality of life and increased societal costs.^2^,^4^ Several treatment interventions have demonstrated large effect sizes for PTSD and comorbid symptom reduction^5^,^7^ and consequently are recommended as treatments of choice for PTSD. These include eye movement desensitisation and reprocessing (EMDR) therapy and various forms of trauma-focused cognitive-behavioural therapy (e.g., prolonged exposure (PE), cognitive (processing) therapy, brief eclectic psychotherapy for PTSD (BEPP), narrative exposure therapy (NET) and imagery rescripting (ImRs)).

With regards to session amount and frequency, treatment guidelines for PTSD advise a minimum of eight sessions of evidence-based treatments, that are delivered at least once a week.^8 9^ Although effective, the standard frequency of spaced (i.e., weekly) trauma-focused treatment (S-TFT) has several drawbacks. Treatment completion typically takes 8–12 weeks, with a longer treatment duration for those who have been exposed to multiple or prolonged traumatic events.^9^ Pooled drop-out rates are relatively high, around 16%,^7^ and are related to (among other factors) treatment dissatisfaction and concerns or constraints interfering with therapy, such as irregular working hours.^10 11^

In massed trauma-focused treatment (M-TFT), time between sessions is strongly reduced, with frequencies ranging from three sessions per week to multiple sessions per day. With less potential for therapy disruption, M-TFT might benefit patients with outside constraints interfering with therapy.^11 12^ Also, M-TFT might enable those with avoidant coping to more easily complete treatment. Randomised controlled trials (RCTs) found that M-TFT leads to faster symptom reduction while maintaining effectiveness in the short term and long term and has drop-out rates either comparable with or lower than spaced treatments.^13^,^16^

Even though M-TFT has potential benefits over S-TFT, the current Dutch treatment guidelines for PTSD only recommend M-TFT after patients have shown an insufficient treatment response to two evidence-based interventions of S-TFT.^8^ Consequently, treatment may become unnecessarily prolonged and cost-ineffective, especially in multiply traumatised patients. A previous study comparing the cost-effectiveness of M-TFT and S-TFT in patients with childhood abuse-related PTSD^17^ found a small but significant difference of 0.027 in quality-adjusted life years (QALYs) in favour of massed treatment. As PTSD results in an average of 15.2 additional days of absenteeism,^3^ the cost-effectiveness of M-TFT might be higher in employed patients due to a faster reduction in absenteeism. Conducting M-TFT as a first-line treatment for PTSD might therefore reduce societal costs related to PTSD.

This paper describes the study protocol for a multicentre, parallel (1:1), single blinded, RCT of M-TFT versus S-TFT as First-Line Intervention for PTSD - Intensive Treatment, using the acronym FLIP-IT. The primary aim of the trial is twofold: (1) to investigate whether M-TFT is non-inferior to S-TFT in reducing PTSD symptoms in employed patients who have been exposed to multiple potentially traumatic events (PTEs) and who seek first-line treatment for PTSD and (2) to investigate the clinical and cost-effectiveness of M-TFT versus S-TFT. Following the literature, we hypothesise that while long-term effectiveness is comparable to S-TFT, M-TFT’s higher session frequency leads to faster symptom relief,^13^,^16^ enhancing patients’ quality of life and leading to lower societal costs.^17^

Secondary aims concern investigating differential effects on drop-out and identifying predictors and moderators of treatment outcome. We hypothesise that M-TFT has lower drop-out than S-TFT. In line with previous research, we also hypothesise that comorbid disorders, dissociation, experiential avoidance, verbal memory, trauma background and cortisol levels are negative predictors of treatment outcome in both conditions, while social support is a positive predictor.^2^ Last, through qualitative analysis, we aim to understand who may benefit most from M-TFT versus S-TFT and how to improve its application.

## Methods and analysis

### Participants

For study eligibility, participants must meet the following inclusion criteria: (1) be 18 years or older; (2) have a PTSD diagnosis according to the Diagnostic and Statistical Manual of Mental Disorders Fifth Edition (DSM-5) as assessed with the Clinician-Administered PTSD Scale – Revised (CAPS-5-R; see below); (3) have a history of exposure to two or more PTEs; (4) have attended less than eight TFT sessions; (5) be employed (working or on sick leave for <2 years). Patients who meet any of the following criteria will be excluded from participation: (1) have a current psychotic disorder, severe alcohol or substance use disorder or high suicidal intent with a concrete suicide plan as assessed through the Mini International Neuropsychiatric Interview – Short (MINI-S, see below) for DSM-5 or severe aggressive behaviour that poses a danger to others (assessed through self-report of aggressive behaviour towards other people in the last month); (2) have an insufficient command of the Dutch language to complete the assessments. The inclusion of 186 participants started on 18 November 2024 and is planned to last until July 2027.

### Study design and procedures

#### M-TFT protocol

The M-TFT protocol is a comprehensive treatment approach designed to address PTSD through a structured series of sessions. Participants engage in two preparatory sessions once per week, lasting 50 min each, focusing on psychoeducation and case conceptualisation. In the third week, the core treatment starts, consisting of 5 days of M-TFT delivered over 1 week or 2 weeks. Each M-TFT day features a 60-minute PE therapy session, a break and a 60 min EMDR session. The sequencing of PE followed by EMDR has been demonstrated to be more effective than the reverse order.^18^ Two 50-minute closing sessions, one in week 5 and one in week 6, consist of summarising learnt information and preventing relapse. To enhance feasibility, therapist rotation may be incorporated into the treatment plan, which has been found to have no negative effects on the therapeutic relationship.^19^ The total treatment duration for M-TFT is 800 min over 5–6 weeks.

#### S-TFT protocol

The S-TFT protocol comprises either 12 weekly 60-minute sessions plus a last 80-minute session (13 total sessions) or 16 weekly 50-minute sessions, both amounting to a total treatment duration of 800 min. This variable session structure was chosen in order to align with the existing financial reimbursement structures of the participating treatment sites. The S-TFT protocol consists of evidence-based first-line treatment interventions according to the Dutch treatment guideline for PTSD,^8^ which include PE, EMDR therapy, BEPP, NET and ImRs. Although different trauma-focused interventions are applied, they share core elements, including psychoeducation, imaginal exposure, emotional processing, cognitive restructuring and/or meaning-making.^20^ This condition therefore strongly resembles standard clinical care in the Netherlands. The S-TFT protocol does not include therapist rotation.

Both treatment conditions will be conducted face to face at five outpatient treatment centres specialising in psychotrauma in the Netherlands: ARQ Centrum’45, ARQ IVP, Psy-Zo!, Psychotraumacentrum Haarlem and the Military Mental Health Care.

We increase treatment fidelity through several measures. Study therapists must be licensed psychologists or be supervised by one. For all treatment interventions, certified and specific training in each modality is required. Also, therapists are required to follow a mandatory 2.5-day certified training on massed trauma treatment, covering the latest developments in the treatment interventions used in the study. Supervision with a frequency of once every 3 weeks for M-TFT and once every 6 weeks for S-TFT is provided to address treatment progress and problems. Furthermore, audio recordings of the therapy sessions will be made, and a random sample of recordings will be checked for treatment fidelity using checklists tailored to the specific treatment.

#### Medication use

Patients are requested to keep their medication type and dosage stable during the study. At the beginning of each treatment day, therapists will inquire about medication type or dosage changes. Adherence to the instruction will also be monitored at all post-assessments and changes will be recorded.

No concomitant TFT during the study is allowed. If patients decide to drop out of the study, they can finish their treatment.

### Measures

Assessments will be conducted at baseline (T0), after massed treatment is finished (7 weeks; T1), after spaced treatment is finished (17 weeks; T2) and at 6 months and 9 months after baseline (T3 and T4). Assessment points are the same for both groups. Table 1 provides an overview of all measures and treatment duration at each timepoint. Primary outcomes consist of PTSD symptom severity and cost-effectiveness (based on quality of life and inventory of societal costs).

**Table 1:** Assessments at each time point

TimepointsAssessments	T00 weeks	T17 weeks(end M-TFT)	T217 weeks(end S-TFT)	T36 months	T49 months
MINI-S	X				
LEC-5	X				
CAPS-5-R	X	X	X	X	X
TIC-P	X		X	X	X
VLGT	X		X		
ITQ	X	X	X	X	X
ACE-IQ	X				
MHQoL	X	X	X	X	X
EQ-5D-5L	X	X	X	X	X
HADS	X	X	X	X	X
MIOS	X	X	X	X	X
AAQ-II	X	X	X	X	X
PID-5	X			X	
DSS-4	X	X	X	X	X
CEQ	X				
CSS	X			X	
Cortisol	X				
CSQ-8 + VAS		X (massed)	X (spaced)		
Treatment format evaluation		X (massed)	X (spaced)		

AAQ-II, Acceptance and Action Questionnaire II; ACE-IQ, Adverse Childhood Experience International Questionnaire for adults; CAPS-5-R, Clinician Administered PTSD Scale for DSM-5 – Revised; CEQ, Credibility and Expectancy Questionnaire; CSQ-8, Client Satisfaction Questionnaire; CSS, Crisis Social Support Scale; DSS-4, Dissociative Symptoms Scale 4; EQ-5D-5L, EuroQol 5 Dimensions scale; HADS, Hospital Anxiety and Depression Scale; ITQ, International Trauma Questionnaire; LEC-5, Life Events Checklist for DSM-5; MHQoL, Mental Health Quality of Life questionnaire; MINI-S, Mini International Neuropsychiatric Interview – Simplified for DSM-5; MIOS, Moral Injury Outcome Scale; PID-5, Personality Inventory for DSM-5-Brief Form-Adult; TIC-P, Treatment Inventory of Costs in Patients with psychiatric disorders; VAS, Visual Analogue Scale; VLGT, Verbal Learning and Memory Test.

#### Clinician-Administered PTSD Scale for DSM-5 – Revised

PTSD symptom severity is assessed based on the index trauma using the Dutch translation of the CAPS-5-R.^21^ The CAPS-5-R is a comprehensive 20-item clinical interview that assesses the presence and severity of the text revised version of the DSM-5 (DSM-5-TR) PTSD diagnostic criteria on an eleven-point scale. Total scores range from 0 to 200, with higher scores denoting greater symptom severity. The CAPS-5-R shows excellent reliability and good convergent and discriminant validity.^21^,^23^

#### EuroQol 5 Dimensions scale

Quality of life is assessed using the EuroQol 5 Dimensions scale (EQ-5D-5L).^24^ This scale comprises 14 items categorised into various aspects of health status rated on a three-point scale. It also includes a 0–100 Visual Analogue Scale (VAS) assessing perceived health and demographic characteristics. The resulting 5-digit index represents a health profile that can be converted into a total score. The EQ-5D-5L has excellent psychometric properties across diverse populations, conditions and settings.^24 25^

#### Mental Health Quality of Life Questionnaire

The Mental Health Quality of Life Questionnaire (MHQoL)^26^ assesses quality of life among individuals with mental health issues. The MHQoL comprises a descriptive system (the MHQoL-7D) and a VAS (MHQoL-VAS) ranging from 0 (‘worst imaginable psychological well-being’) to 10 (‘best imaginable psychological well-being’).^26^ The MHQoL-7D index score can range from 0 to 21, with higher scores indicating a higher quality of life. The MHQoL-VAS is used to record respondents’ self-assessed, overall psychological well-being. The MHQoL exhibits strong reliability and robust construct validity.^27^

#### Treatment inventory of costs in patients with psychiatric disorders

The treatment inventory of costs in patients with psychiatric disorders (TiC-P) maps the costs arising from psychosocial problems in adults.^28^ It consists of three components: general questions, questions regarding medical care utilisation and work-related questions (19 in total). The TiC-P is reliable and has satisfactory construct validity.^28^

### Secondary outcomes

To investigate trauma exposure, comorbidity, experiential avoidance, dissociation, social support, verbal memory and cortisol as predictors and/or moderators of treatment outcome, we will use the Dutch versions of the:

- International Trauma Questionnaire (for complex PTSD).^29 30^
- Moral Injury Outcome Scale (for moral injury after a potentially morally injurious event).^31^
- The Hospital Anxiety and Depression Scale (for comorbid anxiety and depression).^32^
- Avoidance Assessment Questionnaire (for experiential avoidance).^33^
- Personality Inventory for DSM-5-Brief Form-Adult (for maladaptive personality traits).^34^
- Dissociative Symptoms Scale (for dissociative symptoms).^35 36^
- Crisis Support Scale (for social support and satisfaction with support).^37^
- California Verbal Learning Test (in Dutch: Verbal Learning and Memory Test (VLGT); for verbal memory performance).^38 39^
- MINI International Neuropsychiatric Interview – Simplified for DSM-5 (MINI-S; for the exclusion criteria of current psychotic disorder, severe alcohol or substance use disorder, high suicidal intent with a concrete suicide plan).^40 41^
- The Life Events Checklist (LEC-5; for PTEs).^42^
- The Adverse Childhood Events – International Questionnaire (for adverse experiences in childhood).^43^,^45^
- The Credibility and Expectancy Questionnaire (for patients’ confidence in the treatment and perceived treatment credibility).^46 47^

All these instruments have good psychometric properties. Furthermore, we will assess harms as recommended by the STRONG STAR consortium^48^ and hair cortisol for stress levels at baseline.

#### Treatment evaluation

To gain a better understanding of when to implement massed treatment and how to improve its application, we will carry out a qualitative assessment of participants’ experience, consisting of two parts. The first part entails open-ended questions to assess satisfaction with the therapy format and its execution. Participants will answer the questions in table 2 as part of a self-report questionnaire after their treatment has ended (at T1 for the M-TFT condition and at T2 for the S-TFT condition).

**Table 2:** Topic list used in treatment evaluation

Questionnaire after treatment completion	Focus group
What was your experience of the treatment?	What was your experience of the treatment?
What do you see as the advantages of spaced therapy?	Which type (massed or spaced) of therapy do you prefer? Why?
What do you see as the disadvantages of spaced therapy?	What would be the optimal frequency of therapy for you? Why?
What do you see as the advantages of massed therapy?	What factors aided or hindered your treatment?
What do you see as the disadvantages of massed therapy?	What changes can we make to improve the quality of treatment?
For you specifically, what do you think would be the biggest difference between doing massed and spaced therapy?	On which aspects would you have liked to receive more help? On which ones less?
After the treatment, what treatment type would have been a better fit for you? Why do you think that is?	What were some things covered in therapy that were not valuable for you? What were some things covered in therapy that were particularly valuable to you?
What would have been the optimal frequency of treatment for you? Why?	Is there anything else you would like to say about the therapy and how it was given?
Is there anything else you would like to say about the therapy and how it was given?	
*For massed specifically:* what was your experience with therapy rotation?	
*For massed specifically:* what did you think about the order (i.e., PE and then EMDR) of treatments? Would you rather have had it the other way round? Why (not)?	

Note: the interview questions will be asked at T1 for M-TFT and at T2 for S-TFT. Focus groups will be held at the end of the study.

EMDR, eye movement desensitisation and reprocessing; M-TFT, massed trauma-focused treatment ; PE, prolonged exposure; S-TFT, spaced trauma-focused treatment ; T1, 7 weeks after baseline; T2, 17 weeks after baseline.

The second part consists of focus groups with several participants, discussing the prompts in table 2. At least two research team members will identify themes across the entries for each question of the interviews and focus groups. After reaching a consensus for the most applicable themes, each line of text is given a theme code. If entries are inconsistent or rater disagreement exists, they will be flagged and resolved through discussion among the raters. If necessary, a third rater will be involved. After this, a summary of findings will be written for each theme, including each subtheme and elaborated with quotes.

### Sample size calculation

**Figure 1:**
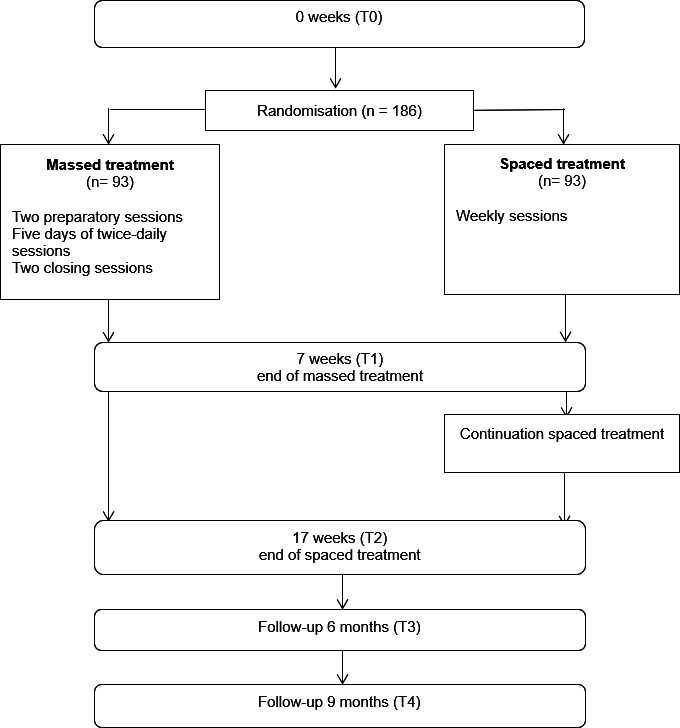
Study design. The design of the study, showing the measurement points and the conditions.

The sample size calculation is based on the upper endpoint of the 95% CI for the difference in mean change scores from baseline to 9 months between M-TFT and S-TFT (adjusted for baseline) being less than the margin of seven CAPS-5 points (not the revised version). As the CAPS-5-R is backwards compatible with the CAPS-5,^22^ we drew from the extant RCT data examining evidence-based PTSD treatment using the CAPS-5. These treatments vary in their minimal change range from 7 to 10 CAPS-5 points, with an SD of 15.6 points.^13^ With a 5% significance level and 80% power, a total sample of 124 participants (62 in both conditions) is needed to detect non-inferiority when M-TFT is truly non-inferior to S-TFT using a 7-point difference on CAPS-5. The sample size was increased to 186, based on the 33% expected drop-out at the last assessment point at 9 months (figure 1).

### Study procedures

The baseline measurement will be split to reduce the burden on participants. One part of the baseline measurement will be conducted immediately after obtaining informed consent (i.e., general questions, inclusion and exclusion criteria, MINI-S, LEC-5, CAPS-5-R, VLGT and hair sample; see table 1). The self-report questionnaires of the baseline measurement are filled out separately, at home, with a research team member present online for questions and completeness of the data.

Consequently, participants are randomly assigned to either M-TFT or S-TFT in a parallel design. The random assignment is carried out on a 1:1 basis using Castor EDC, which incorporates blocked randomisation of 2, 4 and 6, stratified by location. A research associate employed by the sponsor who is not otherwise involved in the study will assign patients to their respective treatment.

Informed consent and the first part of the baseline measurement will take place at a participating centre; all other assessments will take place in online meetings (T1–T4). Assessments will be conducted by psychologists and master’s-level psychology students who have received training in all assessment procedures and instruments. Outcome assessors will administer all clinician-rated instruments blind to treatment conditions. To enhance participant retention, participants will receive €25 if they complete the measurement at T4.

### Analyses

#### Descriptive statistics

We will provide descriptive statistics including demographic variables (age, gender, education level and country of origin) and trauma-related variables (number of PTEs and trauma background). Variables will be tested for differences between treatment completers and drop-outs.

#### Clinical effectiveness

For the primary clinical outcome, repeated measurement analyses using linear mixed models (LMM) will be used to study changes over time in clinician-rated PTSD symptom severity as measured with the CAPS-5-R. We will assess differences in response patterns between the groups. In addition, we will test the difference between the two groups by running a customised test within the LMM at the 9-month time point and base our decision to declare non-inferiority on the corresponding 95% CI. The linear mixed model will compute estimates for missing data. In addition, disaggregated analyses of the primary outcomes will be conducted for gender and trauma background (profession-related vs other). All the analyses above will be carried out on an intent-to-treat basis.

#### Cost-effectiveness analysis

A cost-utility analysis (CUA) will be performed. Cost-utility will be calculated as the difference in mean costs and the difference in QALYs between treatments, yielding a cost per QALY estimate. Societal costs will be valued using reference prices from the Dutch manual for costing in economic evaluations.^49^ Societal costs and QALYs will be modelled for the CUA using generalised linear models (GLM). In addition to calculating the incremental cost-utility ratio (ICUR), an incremental net benefit analysis will be conducted. This approach directly incorporates willingness-to-pay (WTP) thresholds to estimate the net benefit for each treatment. Probabilistic sensitivity analyses will be performed and uncertainty will be visualised using cost-effectiveness acceptability curves (CEACs), which plot the probability that a treatment is cost-effective across a range of WTP thresholds. SEs around the GLM coefficients will be used to explore uncertainty using non-parametric bootstrapping. Cholesky decomposition will be applied to retain the correlations between parameters. Results will be presented in the cost-effectiveness plane and through CEACs to estimate the probability that one treatment is more cost-effective.

### Secondary analyses

For the secondary study parameters, multiple imputations will be applied to missing data. Hierarchical regression analyses will be performed to examine potential predictors of PTSD treatment outcomes and moderators of the relationship between treatment outcome and condition. For this, we will use the severity of comorbid disorders, dissociation, experiential avoidance, social support, verbal memory, trauma background and cortisol at pretreatment. We will correct for multiple tests.

We also intend to calculate a Personalised Advantage Index (PAI). We will use leave-one-out cross-validation to generate the counterfactual prediction per patient using prognostic and prescriptive variables from moderation analyses and generate the PAI, the magnitude of the predicted difference of receiving the predicted optimal treatment versus the non-optimal treatment.^50^ For the trees for treatment-subgroup interactions, we will use the R package quint.

### Adverse events

Therapists will inquire about adverse events at the start of each treatment day. Adverse events will also be monitored at all post-assessments and followed up by the research team until they have abated or until a stable situation has been reached.

A substantial protocol modification will be notified to the Medical Ethics Review Board through an amendment. Only on approval will we make modifications and upload the new protocol version to the registry (Clinicaltrials.gov and Research with Human Participants). Modifications will then be communicated to relevant parties, such as participants, trial therapists and other researchers.

On expressing willingness to participate, the patient will provide written informed consent in twofold based on the Centrale Commissie Mensgebonden Onderzoek (CCMO) approved format (see online supplemental appendix A – in Dutch), which includes an opt-out option for data use for other research, data storage outside the EU and collecting biological hair samples.

### Data management and monitoring

Data will be pseudonymised as much as possible and stored under secure conditions with restricted access, available to the research team only in compliance with current data storage protocols. All electronic data will be collected in a software solution (Castor EDC) and stored on a secured network with servers within the EU. Data will be stored for 15 years after study completion. The metadata will be placed in a repository. The dataset can be delivered on request for meta-analyses and aggregated analyses based on the abovementioned stipulations and will be anonymised by removing identifiable information. If participants wish to stop participating in the study, data previously collected will be stored for up to 15 years and used for initial analyses.

Because this study does not investigate a product, the study was exempted from involving a data safety monitoring board. During the data collection phase, an independent monitor will perform monitoring at least twice a year.

### Dissemination

The trial’s results will be communicated in several ways. First, several peer-reviewed publications will be published in an Open Access format, with a predetermined range and order of authors. Because of the sensitive nature of the data, patients are asked for optional consent to share their anonymised data with other research projects. Data from the patients who give this optional consent are available from the corresponding author via email on reasonable request and with the requirement that the submitted research proposal and data access agreement must first be approved by ARQ Centrum’45 and Rijksuniversiteit Groningen who are joint owners of the data. Results will be presented in meetings of professional associations. In addition, factsheets and information materials on massed versus spaced treatment will be made available for therapists, referring professionals, patients and family members/caregivers, health insurance companies and employers.

### Patient and public involvement

Multiple patients were involved in the design of the study. A patient panel provides feedback throughout the data collection phase on a variety of aspects, such as feasibility and recruitment strategies.

## Discussion

The main goal of this multicentre, parallel, single blinded, non-inferiority RCT is to investigate whether M-TFT in comparison with S-TFT is non-inferior in reducing PTSD symptoms in patients seeking first-time treatment for PTSD, as well as more cost-effective. Although S-TFT is effective, it may be unnecessarily prolonged and consequently relatively cost-ineffective, especially in multiply traumatised individuals who are employed. M-TFT might lower costs from a societal perspective while retaining effectiveness. Should M-TFT prove to be non-inferior to and/or more cost-effective than S-TFT, it might impact both Dutch and international guidelines for first-line PTSD treatment.^8 9^ Regarding the type of massed treatment, we aim to investigate the effectiveness of combining PE and EMDR. This approach, widely used in the Netherlands and with proven preliminary effectiveness in uncontrolled studies (e.g., a study by Zoet *et al.*^51^), has yet to be compared against an active control group. This RCT is particularly well-designed to assess its effectiveness.

The secondary goal of this study is to investigate differential effects on drop-out and identify predictors and moderators of M-TFT and S-TFT. Several previous RCTs have found that M-TFT results in lower treatment drop-out than S-TFT.^52^ With this RCT, we would like to test whether these findings can be replicated. Qualitative interviews with patients will be performed to further contribute to the identification of factors and subpopulations for whom M-TFT might be especially effective.

### Strengths

There are several strengths of this study. First, by studying a massed format in a previously unstudied PTSD population (i.e., those without prior treatment and still employed), we can test whether previous findings of both clinical and cost-effectiveness may be replicated in this new population. Second, the variety of centres throughout the Netherlands ensures a diverse population affected by a broad range of PTE types, which increases the generalisability of findings. Third, by using multiple types of treatment interventions, the control condition strongly resembles standard clinical care. The RCT contains elements of pragmatic trials, therefore introducing more variation between conditions, but also enabling a more valid comparison.

### Limitations

The study design also has several limitations. First, multiple differences between conditions exist. Therapist rotation is only applied in M-TFT. Because therapist rotation is not associated with a significant difference in the therapeutic relationship,^19^ it probably will not explain potential differences in clinical effectiveness. Second, although external validity is increased by allowing multiple treatment interventions, this introduces another confounding variable. However, the treatment interventions share many elements, including psychoeducation, imaginal exposure, emotional processing, cognitive restructuring and/or meaning-making,^20^ keeping variation limited. Third, supervision frequency is higher in M-TFT than S-TFT. This difference in supervision is proportionate to the increased treatment session frequency, affording therapists in both conditions two opportunities per patient.

## Ethics and dissemination

The study procedures were reviewed and approved by the Medical Ethics Review Board of the Amsterdam University Medical Centre (NL86057.018.24), and the protocol (version 3; 16-07-2024) is preregistered in Overview of Medical Research in the Netherlands (OMON; trial register number 56960) and ClinicalTrials.gov (NCT06700590). The sponsor has insurance for compensation to those who suffer harm from trial participation.

## Supplementary material

10.1136/bmjopen-2025-102530 online supplemental appendix 1
