# Supplementary material for: Cost-effectiveness of massed versus spaced trauma-focused treatment as first-line treatment for post-traumatic stress disorder in adults with multiple trauma exposure: protocol for a single-blind non-inferiority randomised controlled trial
Source: BMJ Open. 2025 May 23;15(5):e102530. doi: 10.1136/bmjopen-2025-102530 (PMC12104943; doi:10.1136/bmjopen-2025-102530)
Supplement: online supplemental appendix 1 [file bmjopen-15-5-s001.docx]

**Subject information for participation in medical research**

**Effects and costs of intensive trauma-focused treatment as first-time treatment for people with multiple traumas: the FLIP-IT study**

**Introduction**

Dear Sir/Madam,

With this letter, we would like to ask you to take part in a medical study. Participation is voluntary. You have received this letter because you will soon be undergoing treatment for post-traumatic stress disorder (PTSD). In this letter you will find information about the medical study, what it means for you, and what the pros and cons are. Please take the time to read the letter and to decide whether you would like to take part. If you decide to take part, you will find the consent form in appendix III.

**Ask your questions**

You can take your decision based on the information in this information sheet. We also suggest that you do this:

- Put your questions to the investigator who gave you this information.
- Talk to your partner, family or friends about this study.
- Read the information on [www.rijksoverheid.nl/mensenonderzoek](http://www.rijksoverheid.nl/mensenonderzoek).

1. **General information**

ARQ National Psychotrauma Centre has initiated this medical study. The assessments and treatments will take place at different healthcare institutions, namely Psy-Zo!, ARQ IVP, ARQ Centrum’45, Psychotraumacentrum Haarlem and the Military Mental Healthcare. Participants in a medical study are often called subjects. A subject can be healthy as well as a patient. For this specific study, we need 186 participants from the Netherlands. The study is approved by the Medical Ethics Review Committee of the Amsterdam UMC.

1. **What is the purpose of the study?**

In this medical study we want to investigate whether an intensive treatment for PTSD is as effective as the regular treatment for PTSD. We also investigate whether the intensive treatment costs less.

1. **What is the background of the study?**

The usual treatment for PTSD is once a week, but sometimes a more intensive treatment is used where multiple sessions per week or even per day are offered. Until now the intensive treatment is only used when usual treatment of PTSD does not sufficiently help. In this study some people will receive the intensive treatment as their first treatment. We want to investigate whether it provides similar results as the usual treatment for PTSD. Also, we investigate the costs of the treatment.

1. **What happens during the study?**

**What treatments will be investigated?**

As mentioned, we are comparing two treatments for PTSD in this medical study. Both treatments are described in table 1.

**Tabel 1**

|  | **Spaced trauma-focused treatment (S-TFT)** | **Intensive trauma-focused treatment (I-TFT)** |
| --- | --- | --- |
| **Treatments** | One of these treatments:   - Imaginary exposure with exposure in vivo - EMDR therapy - Narrative Exposure Therapy - Brief Eclectic Psychotherapy for Posttraumatic stress disorder (BEPP) - Imagery rescripting | A combination:   - Imaginary exposure with exposure in vivo (morning) - EMDR therapy (afternoon) |
| **Duration** | Weekly 50 minute treatment during 16 weeks (ARQ IVP) or weekly 60 minute treatment during 13 weeks at Psy-zo!, ARQ C’45, and Psychotraumacentrum Haarlem, of which the last session lasts 80 minutes. In total you will receive 800 minutes of treatment regardless of the centre. | Two trauma-focused treatments per day of 60 minutes each, within a maximum of 6 weeks |
| **Therapists** | 1 therapist | 1 or multiple therapists |

**What do these treatments entail?**

All treatments are evidence-based treatments for PTSD. The treatments are described in table 2.

**Table 2**

| **Imaginary exposure with exposure in vivo** means that the therapist motivates you to repeatedly and extensively relive unpleasant memories of the shocking events. You will be supported to relive the traumatic memories such that processing can take place. During the exposure in vivo you will practise confonting trauma-related events that evoke fear, such as certain objects, people and situations that you would rather avoid since the traumatic experience. During this exercise, you will also be asked about your fearful expectations, which allows you to correct these. This will make you less fearful and you will no longer need to avoid. |
| --- |
| In **EMDR therapy** you will bring the memory to the traumatic event to mind and you will focus on the image that currently still elicits the most distress while you will perform a strongly distracting task. This will reduce the vividness and distress of the traumatic memory and change the meaning. |
| In **Narrative Exposure Therapy** the life history is central. Both the beautiful and the shocking events and periods in between will be placed in the life history and will be treated with exposure therapy. They will become better integrated in your memory and the connection between events becomes clearer. At the end, you can take home the report of your life history. |
| **Brief Eclectic Psychotherapy for Posttraumatic stress disorder (BEPP)** helps you to invite, express and process suppressed, painful feelings and thoughts that are connected to the traumatic events. This happens through exposure and writing assignments. In the second part, the impact of the traumatic events on your life is discussed and there is time for meaning making and a farewell ritual. |
| **Imagery rescripting** is a method where the therapist helps you to look at traumatic memories from your past in a different way which changes the meaning of that experience. Within this method you will, as it were, rescript your memory, together with the therapist. |

**The start: the intake**

After enrollment, we will plan an intake. There, you will discuss the medical study with the researcher. If you want to take part, the researcher will call you after a week to give you some time to think about participating. In the next meeting, you can sign the informed consent form (see appendix III), we will administer interviews and you will fill in questionnaires. This lasts about three hours.

**Are you eligible to take part?**

First, we want to know if you are eligible to take part:

- You are 18 years or older.
- You have PTSD due to two or more traumatic experiences.
- You will receive treatment for PTSD for the first time.
- You are employed (or on sick leave for less than 2 years).
- You do not have serious additional problems that could prevent the treatment of PTSD.

If you want to and are you eligible to take part, you will be randomly assigned to one of the treatment conditions. This means that you cannot choose one of the treatment conditions.

1. **The measurements and agreements during the research**

We want the study to go well. That is why we want to make the following agreements with you. We expect you to come to all of the assessments, apart from the treatment of the treatment. The assessments will take place before the start of the treatment, after seven weeks, seventeen weeks, six months and nine months after the start of the treatment. During the assessments we will administer interviews and questionnaires. These measurements will usually take 1.5 to 2 hours. We will look at your PTSD-symptoms, your quality of life and other complaints, such as anxiety, depression and memory complaints. To investigate your physical response to stress we would also like to measure the stress hormone cortisol. For that we would like to cut a small hair sample once, if you consent. This small hair sample will be used to measure your cortisol levels in a laboratory. After that, your hair sample will be destroyed. If you do not want to do this, you can still participate in the study. The treatment sessions will be recorded and listened to, to ensure that your treatment was of high quality.

It is important to realize that an intensive treatment is a considerable investment. We prefer if you do not use alcohol or drugs during the treatment. Try to not change the dose or type of medication during treatment.

**What is extra or different than the usual treatment you will receive?**

When you are enrolled in the intensive treatment, your treatment will take six weeks instead of the regular 13 or 16 weeks.

1. **What side effects, adverse effects or discomforts could you experience?**

The safety of trauma-focused treatment has been extensively investigated. Because you will be exposed to your traumatic memories during the trauma-focused treatment, you can temporarily experience more complaints lasting several hours to several days. This is true for both treatments. Should you experience a longer lasting period of increased complaints, you can contact your therapist and the treating doctor.

1. **What are the pros and cons if you take part in the study?**

A pro of participating in the study could be that your complaints might decrease faster in the intensive treatment due to the shorter treatment time. With both treatments your therapist will be trained thoroughly in the treatments of this study. The research potentially delivers information that is useful in the treatment of people with PTSD. A con is that the research takes up some of your time. The planning of an intensive treatment might be harder for you than the planning of the weekly treatment, while others might find it easier.

You decide for yourself whether you wish to participate in the medical study or not. If you decide not to participate, you do not need to do anything. You also do not need to explain why you do not want to participate. You will receive the treatment you would receive otherwise.

1. **When does the study end?**

The study will stop after nine months, when you decide to stop, or when the investigator ends the study, where there are no risks for your health with a premature ending. One of the following agencies can decide that the research should stop: ARQ National Psychotrauma Centre, the government or the medical-ethical research committee that evaluates the study.

*What happens if you stop participating in the study?*

The investigators use the data and the results of the analysis of the small hair sample that have been collected up to the moment that you decide to stop participating in the study. If you decide to end your participation, you may request that the hairs be destroyed. Please let the investigator know.

You can finish your treatment within the treatment centre. You can also decide to seek treatment with a different therapist or a different treatment centre. You do not need to participate in the assessments if you do not want to.

*Will you be informed of relevant information of the medical study during your participation?*

Although the medical study was planned carefully, circumstances can change due to your psychological reactions or new information. In that case, we shall discuss this with you immediately, and you will have the choice to continue or to stop. Should your health or safety be at risk, the study will be terminated immediately.

*Will you get the results of the study?*

About 12 months after the study has ended, the investigator will inform you about the most important results of the study may be found on the website of ZonMw.

1. **What will be done with your data** **and body material (the small hair sample)?**

Are you taking part in the study? Then you also give your consent to collect, use and store your data and body material. We also ask you to consent to donate a small hair sample. If you do not consent, you can still take part. If you do consent, you also give consent to collect, use and store the results of the analysis of the small hair sample.

We store the following data: name, gender, nationality, address, date of birth, data about your health, (medical) data that we collect during the study and the outcomes of the analysis of the small hair sample. The hair sample will be destroyed after the analysis. We want to know your nationality because previous research shows that this might influence your treatment and the memory test (if Dutch is not your native language).

*Why do we collect, use and store your data and body material?*

We collect, use and store your data and your body material to answer the questions of this study. And to be able to publish the results. Data and/or body material can be used by ARQ National Psychotrauma Centre and companies that help ARQ National Psychotrauma Centre with the performance of the study, analyzing the results and measurements of the body material.

*How do we protect your privacy?*

To protect your privacy, we give a code to your data and your body material. We only put this code on your data and body material. We keep the key to the code in a safe place in ARQ National Psychotrauma Centre and the Rijksuniversiteit Groningen. When we process your data and body material, we always use only that code. Even in reports and publications about the study, nobody will be able to see that it was about you.

*Who can see your data?*

Some people can see your name and other personal information without a code. This could include data specifically collected for this study, but also data from your medical file.

These are people who check whether the investigators carry out the study properly and reliably. These persons can access your data:

- An auditor who works for ARQ National Psychotrauma Centre
- National and international supervisory authorities, such as the Health and Youth Inspectorate

These people will keep your information confidential. We ask you to give permission for this access. The Health and Youth Inspectorate can access your personal information without your permission.

*For how long do we store your data and body material?*

We store your data ARQ National Psychotrauma Centre and the Rijksuniversiteit Groningen for 15 years. These coded data are protected to guarantee your privacy.

The body material will be destroyed after the analysis. The results of the analysis of your body material will be stored at ARQ National Psychotrauma Centre for 15 years.

*Can we use your data and body material for other research?*

Your collected data and the results of the analysis your body material may also be important for other medical research on PTSD and the further development of trauma-focused treatments. For this purpose, your data will be stored for 15 years. Please indicate in the consent form whether you agree with this. Do you not want to give your consent? Then you can still take part in this study. You will get the same care.

*What happens if there are coincidental findings?*

It is possible that during the study we discover something that is not directly relevant to the study but is important to your health or to the health of your family members. In that case, the investigator will contact your general practitioner. You will then discuss what needs to be done with your general practitioner. The cost of this will fall under your own insurance policy. With the form, you give consent to inform your general practitioner.

*Can you take back your consent for the use of your data?*

You can take back your consent for the use of your data at any time. Please tell the investigator if you wish to do so. This applies both to the use in this study and to the use in other medical research. But please note: if you take back your consent and the investigators have already collected data for research, they are still allowed to use this information. The investigators will destroy your body material after you take back your consent. But if assessments with your body material have been carried out, the investigator can continue to use the results.

*We send your data to countries outside the European Union*

In this study, we will send your coded data also to countries outside the European Union. The privacy rules of the European Union do not apply in those countries. But your privacy will be protected at a similar level. In the consent form you can indicate whether you consent. Do you not consent? Then you can still participate in this medical study.

*Do you want to know more about your privacy?*

- Do you want to know more about your rights when processing personal data? Visit [www.autoriteitpersoonsgegevens.nl](http://www.autoriteitpersoonsgegevens.nl).
- Do you have questions about your rights? Or do you have a complaint about the processing of your personal data? Please contact the person who is responsible for processing your personal data. For the present, this is:
  - Mirjam Mink-Nijdam, the coordinating researcher for this study at ARQ National Psychotrauma Centre. See Appendix I for contact details and website.
- If you have any complaints about the processing of your personal data, we recommend that you first discuss them with the research team. You can also contact the Data Protection Officer of ARQ National Psychotrauma Centre (e-mail: fg@arq.org). Or you can submit a complaint to the Dutch Data Protection Authority.

*Where can you find more information about the study?*

You can find more information about the study on the following website: zonmw.nl/. After the study, the website may show a summary of the results of this study. You can find the study by clicking this link: https://projecten.zonmw.nl/nl/project/cost-effectiveness-intensive-trauma-focused-treatment- versus-spaced-trauma-focused.

1. **Will your general practitioner and/or specialist be informed of participation?**

If you take part in the medical study, we will inform your general practitioner about taking part in the study, if you consent.

1. **Will you receive compensation if you participate in the study?**

If you receive treatment at Psy-zo!, Psychotraumacentrum Haarlem and ARQ Centrum’45, the costs will be compensated from the standard package of health insurance. You still pay your deductible. If you get treatment at ARQ IVP then your employer pays the treatment. There are no extra costs connected to taking part in the medical study. Neither will you get any compensation if you take part in this study. But you will be paid for your travel expenses related to assessment that are not combined with treatment essions. At the end of all assessments of the research, every participant will receive a small gift worth 25 euros as a token of appreciation.

1. **Are you insured during the study?**

Everyone who takes part in this study is ensured. The insurance company will pay for the damage caused by the study. But not all damage. You can find more information about damage and any exceptions in **Appendix II**. It also says who you can report damage to.

1. **Taking part in a focus group at the end of treatment**

We are inviting you to take part in a focus group discussion where we will discuss the experiences you have had. If you decide to participate, you will receive a compensation of 75 euros (excluding traveling expenses). You can choose between an online session or a session in person. We strive for a minimum of 6 participants, because we believe this to be a good group size to enable discussions.

The focus groups will be led by our research team and recorded for analysis through audio recording. Just as for your other data of this study, these recordings will be protected to ensure your privacy.

1. **Do you have any questions?**

Of course you still have time to think about your participation in the study. You can ask questions about the study of the researchers Ytje van Pelt (e-mail: [ytjevanpelt@psy-zo.nl](mailto:ytjevanpelt@psy-zo.nl), telefoonnummer: 0507502088) and Bram Kemmere (e-mail: [b.kemmere@arq.org](mailto:b.kemmere@arq.org), telefoonnummer 071519500). Ytje van Pelt is available during working hours on Thursday and Friday. Bram Kemmere is available during working hours on Monday until Thursday.

1. **How do you give consent for the study?**

You can first think carefully (at least one week) about this study. After at least one week we plan a new phone call. Then you tell the investigator if you understand the information and if you want to take part or not. If you want to take part, fill in the consent form that you can find with this information sheet. You and the investigator will both get a signed version of this consent form.

Thank you for your attention.

The FLIP-IT Team

Appendices

In the appendices of this information letter you will find:

1. Contact details
2. Information about the insurance
3. Informed consent form

**Appendix I Contact details ARQ National Psychotrauma Centre**

**Researchers:**

Ytje van Pelt, e-mail: ytjevanpelt@psy-zo.nl, phone number: 0507502088, available during working hours on Thursday and Friday.

Bram Kemmere, e-mail: b.kemmere@arq.org, phone number: 071519500, available during working hours on Monday through Friday.

**Independent expert:** Hein van Marle, psychiatrist, e-mail: [h.van.marle@arq.org](mailto:h.van.marle@arq.org), phone number: 0715191500, available during working hours on Wednesday and Friday.

**Complaints:** Complaints officer Joke Romijn, e-mail: [klachtenopvang@arq.org](mailto:klachtenopvang@arq.org), phone number: 0715191500, available on Monday through Friday.

**Data Protection Officer**: Judit Bálint, e-mail: fg@arq.org

**For more information about your rights**: principal investigator Mirjam Mink-Nijdam, e-mail: m.mink@centrum’45.nl, phone number: 0206274974, available during working hours on Monday, Tuesday, Thursday and Friday.

**Appendix II: information about the insurance**

ARQ National Psychotrauma Centre has taken out insurance for everyone who takes part in the study. The insurance pays for the damage you have suffered because you participated in the study. This concerns damage you suffer during the study or within 4 years after you participated in the study. You must report damage to the insurer within 4 years.

The insurance does not cover all damages. Below you will find what damage is not covered. These provisions can be found in the 'Besluit verplichte verzekering bij medisch-wetenschappelijk onderzoek met mensen 2015' ('Medical Research (Human Subjects) Compulsory Insurance Decree 2015'). This decision can be found in the Government Law Gazette (<https://wetten.overheid.nl>).

Have you suffered damage as a result of the study? Please report this to this insurer, with its representative M. Wijnsma.

The insurer of the study is:

Name insurer: HDI Global SE

Address: Postbus 925, 3000AX Rotterdam

Telephone number: 010 40 36 100

Email: info@nl.hdi.global

Policy number: V-056-568-170-1

The insurance pays a maximum €650.000 per person and €5.000.000 for the entire study and €7.500.000 per year for all studies by the same sponsor.

Please note that the insurance does **not** cover the following damage:

- Damage due to a risk about which we have given you information in this sheet. But this does not apply if the risk turned out to be greater than we previously thought. Or if the risk was very unlikely.
- Damage to your health that would also have happened if you had not taken part in the study.
- Damage that happens because you did not follow directions or instructions or did not follow them properly.
- Damage to the health of your children or grandchildren.
- Damage caused by a treatment method that already exists. Or by research into a treatment method that already exists.

**Appendix III: Informed consent form – subject**

Belonging to “Effects and costs of intensive trauma-focused treatment as a first line treatment for people with multiple traumatic experiences: the FLIP-IT Study”

- I have read the information sheet. I was able to ask questions. My questions have been answered well enough. I had enough time to decide if I wanted to take part (a minimum of 1 week).
- I know that taking part is voluntary. I also know that at any time I can decide not to take part in the study. Or to stop taking part. I do not have to explain why.
- I give the investigator consent to inform my doctor/specialist that I am taking part in this study. I give consent to give my doctor or specialist information about accidental discoveries made during the study that are important for my health.
- I give consent to collect and use my data. The investigators only do this to answer the question of this study.
- I know that some people will be able to see all of my data to review the study. These people are mentioned in this information sheet. I give consent to let them see my data for this review.
- I know that my coded data will also be sent to countries outside the European Union where the privacy rules of the European Union do not apply.
- I want to take part in this study.

Please tick yes or no in the table below.

| I give consent to store my data to use for other research, as stated in the information sheet. | Yes ☐ | No☐ |
| --- | --- | --- |
| I give consent to approach me after treatment completion to take part in a focus group, as described in the information form. | Yes ☐ | No☐ |
| I give consent to share my data outside the EU. | Yes ☐ | No☐ |
| I give consent to collect and use my body material (small amount of hair). The investigators only do this to answer the question of this study. | Yes ☐ | No☐ |
| I give consent to ask me after this study if I want to participate in a follow-up study. | Yes ☐ | No☐ |

My name is (subject): ………………………………..

Signature: ……………………… Date : __/__/__

-----------------------------------------------------------------------------------------------------------------

I declare that I have fully informed this subject about the study mentioned.

If any information becomes known during the study that could influence the subject's consent, I will let this subject know in good time.

Investigator name (or their representative): ........................

Signature:……………………… Date: __/__/__

-----------------------------------------------------------------------------------------------------------------

*The study subject will receive a complete information sheet, together with a signed version of the consent form.*
