# Supplementary material for: Cost-effectiveness of massed versus spaced trauma-focused treatment as first-line treatment for post-traumatic stress disorder in adults with multiple trauma exposure: protocol for a single-blind non-inferiority randomised controlled trial
Source: BMJ Open. 2025 May 23;15(5):e102530. doi: 10.1136/bmjopen-2025-102530 (PMC12104943; doi:10.1136/bmjopen-2025-102530)
Supplement: Supplementary file 2 [file bmjopen-2025-102530.reviewer_comments.pdf]

PEER REVIEW HISTORY

BMJ Open publishes all reviews undertaken for accepted manuscripts. Reviewers are asked to complete a checklist review form and are provided with free text boxes to elaborate on their assessment. These free text comments are reproduced below.

ARTICLE DETAILS

Title (Provisional)

Cost-Effectiveness of Massed versus Spaced Trauma-Focused Treatment as First-Line Treatment for Post-Traumatic Stress Disorder in Adults With Multiple Trauma Exposure: Protocol for a Single-blind Noninferiority Randomized Controlled Trial

Authors

Kemmere, Bram; van Pelt, Ytje T.; Lommen, Miriam J. J.; Huntjens, Rafaele J. C.; Olff, Miranda; Méndez, Mayaris Zepeda; Nijdam, Mirjam J.; Matthijssen , Suzy; Hakkaart-van Roijen, Leona; Heide, F. Jackie June ter

---

This article was not externally reviewed at BMJ Open. Protocols that have been independently assessed prior to submission to BMJ Open are usually fast-tracked to publication on the grounds that further substantial changes will not be possible. This independent assessment will usually be external, independent review for both a substantial grant award from a non-commercial or government funder and ethics approval.
